# A word on standardization in longitudinal studies: don't

**DOI:** 10.3389/fpsyg.2015.01389

**Published:** 2015-09-15

**Authors:** Julia Moeller

**Affiliations:** Yale Center for Emotional Intelligence, Yale UniversityNew Haven, CT, USA

**Keywords:** standardization, ipsatization, longitudinal data, profiles, experience sampling method (ESM)

“*Typically, you don't want to do a full z-score standardization of each variable, because then you lose the covariance metric that is needed for the SEM procedures, and you lose any information about mean-level changes over time.”*

*(Little*,*2013**, p. 18)*

This article discusses the risks of standardization and ipsatization in longitudinal studies. First, it summarizes some common purposes of standardization in psychological studies. Second, it explains why and when standardization and ipsatization are problematic in the analysis of longitudinal data and profiles. Third, it shows alternative ways to achieve similar purposes while avoiding the risks.

## Definitions and purposes of Z-standardization and ipsatization

Z-standardization and ipsatization are procedures to transform absolute values, or ratings (e.g., 1 = *don't agree at all* to 7 = *totally agree*) to relative scores that reflect each answer's rank in comparison to the ranks of all responses in that sample. In z-standardization, the sample mean score is subtracted from each single observation, and this difference is then divided by the sample's standard deviation. The result is a scale where a score of 0 means that this observation was at the sample's mean level, and a z-score of 1 reflects an observation one standard deviation above the sample mean. Ipsatization also converts absolute ratings into relative ranks, but relates each answer to the individual's own mean, not the sample mean. An individual's responses are ipsatized by subtracting the individual's mean score from each response the individual gave in a questionnaire. A positive ipsatized score means that the individual rated this item higher (which often means: affirmed more strongly), than the average of other items in that questionnaire.

Standardization and ipsatization are applied for the following purposes:

1. Standardization is used to bring variables with different response scales (e.g., a scale from 1 = *don't agree at all* to 7 = *totally agree* and another from 0 = *don't agree at all* to 10 = *totally agree)* to a comparable metric.
2. Z-standardized scores are displayed in graphs to accentuate the mean-level differences between groups or profiles of observations.
3. Ipsatization is used to account for uniform response biases, such as acquiescence (=tendency to affirm all items). For instance, in cross-cultural comparisons, items are often ipsatized to account for culture-specific response biases (Tweed and DeLongis, 2006). For the same purpose, within-person standardization is applied in intensive longitudinal studies with many observations per person across short time spans (e.g., experience sampling method, see Csikszentmihalyi and Schneider, 2000). While ipsatization refers to the individual's mean score across all variables, intra-individual standardization refers to the individual's mean of one variable across multiple observations. The resulting ipsatized and intra-individual z-scores reflect whether a response was “high” compared to other responses of the same individual. In regression analyses, the predictor variable is often ipsatized at the mean of the sample or group in order to make the intercept meaningfully interpretable (=“centering,” see Enders and Tofighi, 2007).

## Problems arising through standardization and ipsatization

While standardization and ipsatization are easy and widely accepted, there are many constellations in which these procedures are not useful or misleading. For cross-sectional studies, these issues have long been discussed (e.g., Fischer and Milfont, 2010), but additional problems arise when longitudinal data or profiles are examined. The reasons for the additional problems of standardization in longitudinal and nested data are the many additional possibilities to relate ratings to different reference frames and distributions. In longitudinal studies, the questions arise: Shall we standardize within time points, or across them? Standardize within or across individuals? Standardize within or across age groups/cohorts? The resulting problems are:

1. Standardizing repeated measures within individuals impedes examining mean-level differences between individuals, because each individual's mean score becomes zero. The standardized means don't inform whether the individuals differed in their original experiences.
2. Standardization across individuals within measurement time points impedes examining mean level changes from one time point to another, because all means at all time points become zero, whereas the raw-score means might have shown a decrease in the measured variable, such as interest (see e.g., Denissen et al., 2007).
3. Standardization across individuals across time points obfuscates the information about the relative rank of an individual at given time points, and impedes disentangling rank-order and mean-level stability. For instance, Anna might have had relatively high interest in grade one and grade three, compared to others at the same time point. However, since interest often decreases with time, Anna's absolute interest was much lower in grade 3 than grade 1, as was everybody else's. With standardization across time points and individuals, the information about the time-point-specific relative rank-order gets mixed with the mean-level change, and it will look like Anna had high interest at time one but somewhat low or medium interest at time two.
4. Standardization across individuals within age groups/cohorts impedes studying age differences at given time points. For instance, in a study that examined three cohorts (6th, 8th, and 10th grade) in 3 years (1992, 1995, 1997; see Csikszentmihalyi and Schneider, 2000), standardizing across individuals within each cohort or year impedes examining whether a mean score changed from the 6th to 8th grade, or from 1992 to 1995, because they all become zero.
5. Misinterpretation of differences between profiles and groups is likely when z-standardized scores are used to compare these profiles, particularly if the variables differed in their means and variances prior to the transformation. Two problems complicate interpreting group differences based on z-scores: First, the z-scores represent ranks in relation to other individuals, but not the degree to which an item was affirmed by a given individual. If an item had a low sample mean score, then a “high” z-score above 0 (above the sample mean) can represent a “rather not” statement below the midpoint of the original response scale (see Moeller et al., in press). Second, plotting group differences using z-scores often makes eventually small differences look big, compared to a graph displaying the complete original response scale and raw scores. The reason is that z-score-based graphs often show only the part of the distribution where profiles differed, instead of the complete range of possible answers (e.g., Tuominen-Soini et al., 2011). This is similar to a graph with a truncated y-axis, which is considered misleading (e.g., Rovezzi Carroll and Carroll, 2002).
6. Standardization across individuals should not be done with ipsatized scores, because that entangles the intra-individual frame of reference (ipsatization) and the inter-individual frame (standardization) and is hard to interpret.
7. Ipsatization changes the covariance matrix in a way that makes the data unsuitable for correlational techniques like exploratory and confirmatory factor analysis, structural equation modeling, and multivariate techniques like multiple regression and multivariate analysis of variance (Cornwell and Dunlap, 1994; Closs, 1996; Chan, 2003).

Due to the complexity of longitudinal data and analyses, the above-described problems often co-occur. For instance, standardizing situation-specific repeated measures across individuals increases the risk of misinterpreting mean differences of z-scores between situation-level profiles of state measures, because the z-standardized situation-specific measures are at the same time determined by the intra-individual distribution of these variables (see problem no. 5), and the inter-individual distribution of these variables (see problems 2–5). This makes it almost impossible to interpret whether a relatively high rank (z-score) represents a variable that was rated as “high” on the original response scale by a specific person in a specific situation. For an example of intertwined standardization problems, see Denissen et al. (2007), who applied two different standardization strategies (within individuals across measurement time points; and across individuals within time points), and then compared within-time-point profiles of the standardized variables. This strategy includes three risks: those related to standardization within individuals (problem 1), those related to standardization within time points (problems 3 and 4), and those related to misinterpretation of profile mean scores (problem 5).

## Alternatives to standardization and ipsatization

For bringing differently measured items to the same metric, there are several easy alternative monotonous scale transformations available, which, unlike standardization, do not change the multivariate distribution and covariance matrix of the transformed variables. One solution is the proportion of maximum scaling (“POMS”) method (Little, 2013), which transforms each scale to a metric from 0 (=minimal possible) to 1 (=maximum possible), by first making the scale range from 0 to the highest value, and then dividing the scores by the highest value.

POMS=[(observed−minimum)/(maximum−minimum)]

For instance, for a scale that originally ranged from 1 to 7, first the value 1 is subtracted from each observation to make the scale go from 0 to 6, and then each score is divided by 6 to make the scale go from 0 to 1. Contrary to standardization, this maintains the proportions of the absolute distances between the observed response options.

Another possibility is the percent of maximum possible (“POMP”) method (Cohen et al., 1999), which makes each scale range from 0 (=minimal possible) to 100 (=maximum possible) by multiplying the result of the POMS transformation by 100. The resulting POMP-transformed scores can be interpreted as percentages of the possible maximum score. SPSS syntaxes for these transformations can be downloaded freely (Moeller, 2015).

For examining mean-level differences between profiles and groups, raw scores or scales transformed with the POMS or POMP method can be used. This has the advantages that the scores reflect the individual's degree of affirmation/rejection of the items, and that group differences are displayed in the correct proportions. For a discussion of further advantages and alternative transformations, see Little (2013), and Cohen et al. (1999).

To account for uniform response bias such as acquiescence, a common-method factor can be modeled in structural equation models (Billiet and McClendon, 2000; Geiser and Lockhart, 2012). For instance, a latent variable with similar factor loadings on all observed answers in the questionnaire can account for the response tendency that all observed answers had in common. The advantage over ipsatization is that the covariance metric remains useful for all exploratory and confirmatory factor analyses (EFA, CFA), and structural equation modeling (SEM). The procedure can be adapted to account for non-uniform response biases (e.g., combined affirmation bias to positively perceived questions, and negation bias to negatively perceived questions, as relevant in symptom validity assessment). If instead ipsatization is used with EFA, CFA, or SEM, then the ipsatization procedure must be modified (see Chan and Bentler, 1993; Cheung and Chan, 2002). To account for response biases in the analysis of profiles, method factors and raw- or POMS-scores can be combined in factor-mixture models (Lubke and Muthén, 2005; Leite and Cooper, 2010).

With both ipsatization and method factors, it remains difficult to disentangle biased response styles from genuine experiences. For instance, some individuals really are interested in a broad variety of topics (=affirm all interest items) and do not show are clear interest profile with high interest in some and low interest in other topics (Rounds and Tracey, 1993). To disentangle scale usage from genuine experiences, it helps including contradicting items and constructs in the questionnaire, or using validity scales, e.g., to assess the tendency to generally affirm items disregarding their content, or assessing social desirability. The research on “scale usage heterogeneity” provides further tools for this purpose (Rossi et al., 2001, 2005).

## Summary

Z-standardization is a widely used procedure, applied for getting rid of acquiescence and other response biases, bringing variables of different metrics to the same metric, and emphasizing differences between groups in graphs.

In longitudinal data and analyses of subgroups of observations, z-standardization leads to a number of problems. It changes in often undesirable ways the distances between observations, and the multivariate distributions of cross-sectional and longitudinal data. The psychological literature is rich in examples of misinterpreted z-scores, some of which were described in this article. While many pitfalls are known for cross-sectional studies, longitudinal studies add further problems, due to confounded frames of reference (the original response scale, the intra-individual distribution, the inter-individual distribution within given time points, the inter-individual distribution across different time points, the variation within vs. between cohorts, and any combinations of these). Generally, it is not insightful to first standardize variables within units (individuals, cohorts, states, organizations) and then compare mean scores across these units that gave the reference frame for standardization. This should be trivial, but can often be observed in the current research, and is easily overseen or mishandled the more units and reference frames are added to the data structure.

Modeling common-method factors is a useful alternative to account for response biases while avoiding the downsides of ipsatization. Alternative easy monotonous scale transformations are available to get items with different response scales to the same metric (Cohen et al., 1999; Little, 2013). Given the ease and wide acceptance of standardization in the psychological literature, it seems necessary to emphasize the risks and possible misinterpretations during the methodological training, writing and review processes in psychology. As Little (2013) pointed out, it seems wise to avoid standardization in longitudinal data analyses and person-oriented analyses, unless the researcher is fully aware of and able to avoid undesirable consequences. There are many good uses for these procedures, but also many risks.

## Conflict of interest statement

The author declares that the research was conducted in the absence of any commercial or financial relationships that could be construed as a potential conflict of interest.
